# Role of Fibroblast Growth Factor 10 in Mesenchymal Cell Differentiation During Lung Development and Disease

**DOI:** 10.3389/fgene.2018.00545

**Published:** 2018-11-14

**Authors:** Jin Wu, Xuran Chu, Chengshui Chen, Saverio Bellusci

**Affiliations:** ^1^Institute of Life Sciences, Wenzhou University, Wenzhou, China; ^2^Excellence Cluster Cardio-Pulmonary System, Universities of Giessen and Marburg Lung Center, Member of the German Center for Lung Research, Justus-Liebig-University Giessen, Giessen, Germany; ^3^Department of Pulmonary and Critical Care Medicine, The First Affiliated Hospital of Wenzhou Medical University, Wenzhou, China

**Keywords:** Fgf10, lung, lipofibroblasts, alveolar myofibroblasts, fibrosis

## Abstract

During organogenesis and pathogenesis, fibroblast growth factor 10 (Fgf10) regulates mesenchymal cell differentiation in the lung. Different cell types reside in the developing lung mesenchyme. Lineage tracing *in vivo* was used to characterize these cells during development and disease. Fgf10-positive cells in the early lung mesenchyme differentiate into multiple lineages including smooth muscle cells (SMCs), lipofibroblasts (LIFs) as well as other cells, which still remain to be characterized. Fgf10 signaling has been reported to act both in an autocrine and paracrine fashion. Autocrine Fgf10 signaling is important for the differentiation of LIF progenitors. Interestingly, autocrine Fgf10 signaling also controls the differentiation of pre-adipocytes into mature adipocytes. As the mechanism of action of Fgf10 on adipocyte differentiation via the activation of peroxisome proliferator-activated receptor gamma (Pparγ) signaling is quite well established, this knowledge could be instrumental for identifying drugs capable of sustaining LIF differentiation in the context of lung injury. We propose that enhanced LIF differentiation could be associated with improved repair. On the other hand, paracrine signaling is considered to be critical for the differentiation of alveolar epithelial progenitors during development as well as for the maintenance of the alveolar type 2 (AT2) stem cells during homeostasis. Alveolar myofibroblasts (MYFs), which are another type of mesenchymal cells critical for the process of alveologenesis (the last phase of lung development) express high levels of Fgf10 and are also dependent for their formation on Fgf signaling. The characterization of the progenitors of alveolar MYFs as well the mechanisms involved in their differentiation is paramount as these cells are considered to be critical for lung regeneration. Finally, lineage tracing in the context of lung fibrosis demonstrated a reversible differentiation from LIF to “activated” MYF during fibrosis formation and resolution. FGF10 expression in the lungs of idiopathic pulmonary fibrosis (IPF) vs. donors as well as progressive vs. stable IPF patients supports our conclusion that FGF10 deficiency could be causative for IPF progression. The therapeutic application of recombinant human FGF10 is therefore very promising.

## Introduction

Fibroblast growth factor 10 is a member of the Fgf7 subfamily of secreted growth factors. Fgf10 is mostly secreted by the mesenchyme and acts on the epithelium via the Fgfr2b and Fgfr1b receptor (Ornitz and Itoh, 2015). *Fgf10* deletion in mice leads to aborted limb development as well as perinatal lethality due to impaired lung development. This phenotype is shared with *Fgfr2b* knockout embryos indicating that Fgf10 acts mostly via Fgfr2b during organogenesis (Sekine et al., 1999; De Moerlooze et al., 2000).

Fibroblast growth factor 10 also contributes to the formation of the white adipose tissue and the associated mammary gland as well as the heart, liver, brain, kidney, prostate, cecum, ocular and salivary glands, thymus, inner ear, tongue and trachea (Itoh and Ohta, 2014). In the developing lung, *Fgf10* expression is detected at the onset of the pseudoglandular stage (embryonic day (E) 9.5-E16.5), as early as E10, when the primary bronchi are formed. *Fgf10* expression in the distal mesenchyme between E10 and E12.5 coincides with epithelial bud formation suggesting that this growth factor plays a key role during branching morphogenesis. Interestingly, at E10, the rudiments of the two primary bronchi are clearly visible in the lungs of *Fgf10* KO embryos suggesting that Fgf10 is dispensable for the very initial step of lung development involving the formation of the two primary lung buds from the ventral foregut endoderm. At E13.5, Fgf10 expression is found ubiquitously throughout the mesenchyme and its role in guiding the branching process is not clear (Bellusci et al., 1997). Its widespread spatial expression suggests that Fgf10 plays a permissive more than an instructive role during the branching process. It is very likely that other players such as heparan sulfate proteoglycans, which have a high affinity for Fgf10 as well as other growth factors, are interacting with Fgf10 to restrict its activity distally in order to control the branching process. For details on Fgf10 signaling *per se*, we refer to other mini-reviews also published as part of this special issue (Ndlovu et al., 2018; Watson and Francavilla, 2018). During the subsequent stages of mouse development (canalicular: E16.5 to E17.5, saccular: E17.5 to postanatal day (P) 5; alveolar: P5 to P28), Fgf10 is still significantly expressed suggesting that Fgf10 could play multiple roles beyond the pseudoglandular stage not only in the epithelium, but also directly or indirectly in the mesenchyme. This last aspect in particular has been widely ignored, yet may hold the key to potential therapeutic interventions to treat human lung diseases.

## Different Mesenchymal Cell Types Exist in the Developing Lung Mesenchyme

**FIGURE 1:**
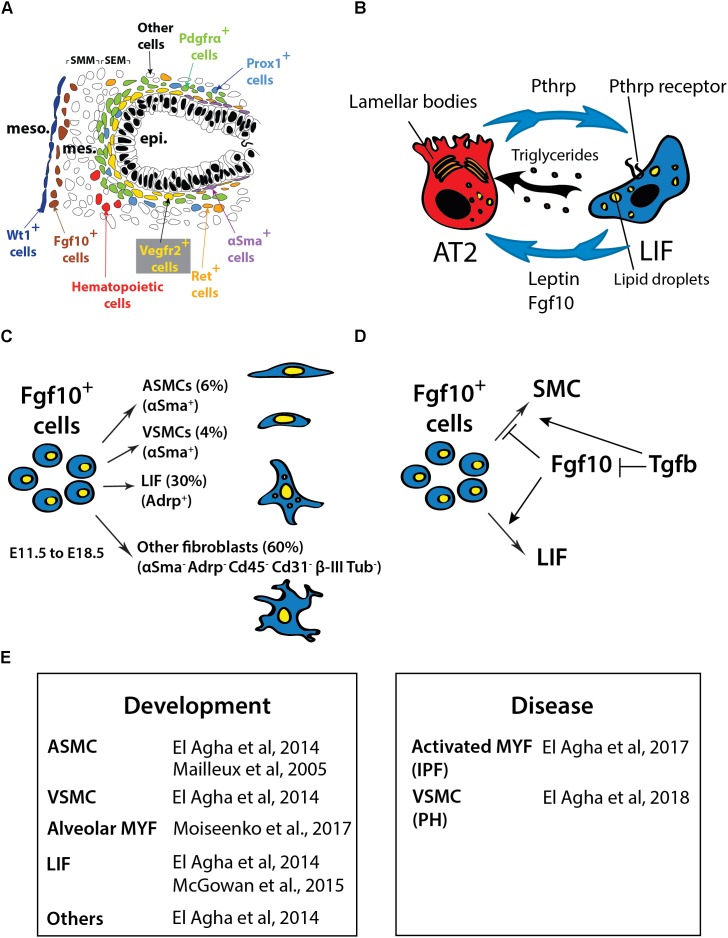
**(A)** Schematic of the cells in the distal part of the lung at E13.5 showing the different lung domains including the mesothelium (meso), the submesothelial mesenchyme (SMM), the subepithelial mesenchyme (SEM), the epithelium (epi) and the position of the different mesenchymal cell progenitors located at this stage. **(B)** Interaction between AT2 and LIF. LIF produce leptin and triglycerides that are essential for surfactant production. The Pthrp/ Pparγ axis is critical for LIF formation and maintenance. **(C)** Fgf10-positive cells lineage-traced at E11.5 and analyzed at E18.5. **(D)** Fgf10 acts directly on the mesenchyme to induce the differentiation of LIF progenitors. Tgfβ1 antagonizes this differentiation (adapted from El Agha et al., 2014; Al Alam et al., 2015; Chao et al., 2015). **(E)** Table summarizing the cell types expressing *Fgf10* in the lung during development and disease. IPF, Idiopathic pulmonary fibrosis; PH, pulmonary hypertension.

The embryonic lung mesenchyme displays many different types of cells such as chondrocytes, airway smooth muscle cells (ASMCs), vascular smooth muscle cells (VSMCs), nerve cells, endothelial cells, lipofibroblasts (LIFs), lymphatic cells, alveolar myofibroblasts (MYFs) and cells that are uncharacterized previously. Altogether these cells play important roles during embryonic development (Figure 1A) as well as homeostasis in the post-natal stages (for more details on this topic please see El Agha et al., 2014).

## Fgf10 Regulates Mesenchymal Cell Differentiation in the Lung

Ramasamy et al. (2007) demonstrated that *Fgf10* hypomorphic embryos (displaying around 20% of the normal *Fgf10* expression) exhibited major defects in different mesenchymal cell types. Those include ASMCs, endothelial cells and alveolar MYFs. As Fgf10 acts mostly on the epithelium via the fibroblast growth factor receptor 2b (Fgfr2b), some of these defects could be due to impaired epithelial to mesenchymal interactions. However, it was also reported that Fgf10 acts directly on the mesenchyme to control the differentiation of LIF progenitors (Al Alam et al., 2015). In the following sections, we will delineate what is known about the formation of the different mesenchymal cell lineages in the lung and further develop the function of Fgf10 in this context.

## Lineage Tracing *In Vivo* Has Been Used to Characterize Different Mesenchymal Lineages During Lung Development

The secondary heart field (SHF), a cell set contributing progressively to the poles of the elongating heart tube during looping morphogenesis, was recently described as a source of multipotent cardiopulmonary progenitors and is identified and defined by the co-expression of *Wnt2, Gli1* and *Isl1* (Peng et al., 2013). These cells migrate into the lung and differentiate into vascular and airway SMCs as well as other lineages. Fate-mapping of platelet derived growth factor receptor beta (Pdgfrβ)-positive cells showed that VSMCs do not arise from mesothelial but rather from mesenchymal progenitor cells (Greif et al., 2012). Two studies using animal models published contradicting results. The first study using the *Wilms tumor 1 homolog* (*Wt1*)*-Cre* transgenic line, showed that the mesothelium contains progenitors for vascular but not airway SMCs (Que et al., 2008). On the other hand, the second study showed, using the inducible *Wt1^Cre-ERT2/+^* knock-in mice, that the mesothelium is a source of airway and vascular SMCs as well as desmin-positive fibroblasts (Dixit et al., 2013). More work will have to be done to clarify the contribution of the mesothelium to the SMC lineages during development. Endothelial progenitors express fetal liver kinase 1 (Flk-1 or Vegfr2) (Yamaguchi et al., 1993; Kappel et al., 1999; del Moral et al., 2006a,b) whereas lymphatic cells arise from prospero homeobox protein 1 (*Prox1*)-positive progenitor cells (Srinivasan et al., 2007). Finally, nerve cells originate from the neural crest and are marked by receptor tyrosine kinase expression (Langsdorf et al., 2011).

## Fgf10-Positive Cells in the Early Lung Mesenchyme Differentiate Into Multiple Lineages

Mailleux et al. (2005) previously utilized the transgenic reporter line *Mlcv1v-nLacZ-24* (or simply *Fgf10^lacZ^*) to demonstrate that Fgf10-positive cells serve as progenitors for ASMCs in the distal lung during early development. These results were validated using an *Fgf10^Cre-ERT2^* knock-in line (El Agha and Bellusci, 2014). Lineage labeled Fgf10-positive cells mainly differentiates into the airway and vascular SMC and the LIF lineages (El Agha et al., 2014; Figures 1C,E). However, it is still unclear whether this population of Fgf10-positive mesenchymal progenitor cells contains unipotent or multipotent progenitor cells. The progeny of Fgf10-expressing cells needs to be analyzed using single-cell transcriptomic approaches as well as multi-color Cre-reporter mice to better characterize these progenitor cells. During the early pseudoglandular stage, Fgf10 itself is not acting on the ASMCs to control their differentiation. It has been proposed that bone morphogenetic protein 4 (Bmp4), which is induced by Fgf10 in the distal epithelium, is responsible for their differentiation (Mailleux et al., 2005). In addition, it has been shown that β-catenin signaling in the mesenchyme does not contribute to the differentiation of Fgf10-positive progenitors but rather to their proliferation (De Langhe et al., 2008). More recently, *miR-142-3p*, a miR that is enriched in the mesenchyme, was reported to target *adenomatous polyposis coli* (*Apc*), a gene encoding a negative regulator of β-catenin. Upregulated *Apc* expression in the lung mesenchyme upon *miR-142* knock down using morpholinos leads to the inhibition of mesenchymal proliferation and premature SMC differentiation. The corresponding loss of mesenchymal β-catenin signaling in the mesenchyme was associated with decreased *Fgfr2c* and *Fgf10* expression (Carraro et al., 2014).

## LIF-Derived Fgf10 and AT2 Stem Cell Homeostasis

Lipofibroblasts are adipocyte-like fibroblasts located close to AT2 stem cells (O’Hare and Sheridan, 1970; Vaccaro and Brody, 1978). It has recently been suggested that LIFs represent a special niche for AT2 stem cells. LIFs are likely important for AT2 stem cell homeostasis and express high levels of *Pparg, Adrp* and *Pthrp receptor* (*PthrpR*) (Habiel and Hogaboam, 2017). LIFs also contribute to the maturation of alveolar epithelial cells and the formation of surfactant, a phospholipoprotein complex produced by AT2 cells involved in the reduction of surface tension (Rehan et al., 2005). The growth of AT2 stem cells in Matrigel to form alveolosphere-like structures is drastically enhanced when co-cultured with LIFs (Barkauskas et al., 2013). The role that LIFs play under these conditions remains unclear. As previously described, LIFs (or at least a subset of them) are derived from Fgf10-positive cells (El Agha and Bellusci, 2014). Interestingly, a significant proportion of the LIFs in the post-natal lungs are positive for *Fgf10* expression (Al Alam et al., 2015). It is possible that Fgf10 secreted by the LIFs is needed for the maintenance of the AT2 stem cells in the adult lung during homeostasis and/or after injury (Figure 1B). This function would then be similar to what is proposed for the role of Fgf10 in the developing lung, where Fgf10 maintains the sex-determining region Y-box 9 (Sox9)-positive multipotent progenitor cells at the tips of the developing lung. In the future, it will be important to characterize the role of Fgf10 in LIF formation/maintenance in the context of the adult AT2 stem cell niche. In addition, a better characterization of the molecular differences among the diverse LIF subpopulations (Fgf10-positive and -negative LIFs) will be important to identify the mesenchymal cell types that play critical roles in repair processes after lung injury.

## Common Molecular Mechanisms Controlling LIF and Adipocyte Formation: a Critical Role for Fgf10 in Controlling Cell Differentiation

Lipofibroblasts and adipocytes require Pparγ, a master regulator of lipogenesis, for their differentiation (Torday et al., 2003). *In vitro* differentiation assays using NIH3T3-L1 pre-adipogenic cells show that insulin and cortisone, which are used to push these cells toward a mature adipocyte phenotype, induce *Fgf10* expression. In this system, use of blocking antibodies against Fgf10 inhibits pre-adipocyte differentiation (Sakaue et al., 2002). *In vivo, Fgf10* is detected both in pre- and mature adipocytes and is critical for white adipose tissue formation (Mailleux et al., 2002; Sakaue et al., 2002). Beyond its role on differentiation, Fgf10 has also been described to increase cell proliferation in the white adipose tissue (Konishi et al., 2006).

At the end of the pseudoglandular stage of lung development (from E15.5 to E16.5), a subpopulation of mesenchymal cells containing lipid vesicles can be detected using LipidTOX, a non-toxic fluorescent dye that has been instrumental to label LIFs for immunofluorescence studies, flow cytometry and sorting (Al Alam et al., 2015). The emergence of these cells is associated with the increase in the expression of *Fgf10, Adrp* and *Pparg* in the mesenchymal compartment. Even though Fgf10-positive LIFs represent only a subset of the total LIF population, the inactivation of Fgfr2b ligands as well as decreased *Fgf10* expression decreases the overall LIF population. Our results indicate that Fgfr1b and Fgfr2b in the mesenchyme, which can both bind Fgf10, play redundant functions in controlling LIF formation. Interestingly, the LIFs found in the P8 lung display high levels of *Fgf10* and its associated receptors *Fgfr1b* and *Fgfr2b* (McGowan and McCoy, 2015) suggesting that Fgf10 may also play a role postnatally.

In the human lung, *FGF10* expression is increased between 10 and 18 weeks of gestation (corresponding to the pseudoglandular stage of lung development) while *ADRP* expression is unchanged between 10 and 21 weeks (the canalicular stage in human is from 17 to 26 weeks of gestation) (Chao et al., 2015), These results suggest that as in mice, the formation of LIFs in humans occurs mostly during the mid-canalicular stage of lung development.

The emerging picture is that Fgf10 signaling is critical for LIF formation during the late phases of lung development. Fgf10 secreted by mesenchymal progenitor cells, as well as Pthrp, a cytokine secreted by the AT2 cells are both capable of triggering Pparγ signaling on LIF progenitors, which is likely important for their differentiation along the LIF lineage, as well as for the maintenance of their differentiation. Indeed, the deletion of *Pthrp* in mice results in impaired alveoleogenesis and deficient surfactant production (Rubin et al., 2004; Torday and Rehan, 2007; Rehan and Torday, 2012; ). LIFs depend on Pparγ signaling to express Adrp, which is required for triglycerides trafficking from LIFs cytosol to adjacent AT2 cells. This process is essential for surfactant production (Schultz et al., 2002). Leptin, which is secreted by LIFs, also acts on AT2 to increase surfactant synthesis (Torday et al., 2002). Additionally, LIFs contain high levels of RA, which has been shown to be critical for alveolar septation (Simon and Mariani, 2007).

During lung development, Tgfβ1 signaling through Alk5 in the mesenchyme is critical to control the cell fate decision between MYFs and LIFs. *Alk5* conditional knockout (CKO) lungs displayed reduced number of Acta2-positive cells and corresponding increase in LIFs. Alk5 signaling directly or indirectly regulates the expression of *Pdgfra, Pparg, pair related homeobox 1* (*Prrx1*) and z*inc finger protein 343* (*Zfp423*) (Li et al., 2016). As Tgfβ1 and Fgf10 signaling pathways antagonize each other (McQualter et al., 2013) (Figure 1D), it is proposed that the loss of Tgfβ1 signaling in the mesenchyme allows enhanced Fgf10 signaling in the same cellular compartment thereby leading to increased LIF formation.

## Alveolar MYF and Pdgfa Signaling

**FIGURE 2:**
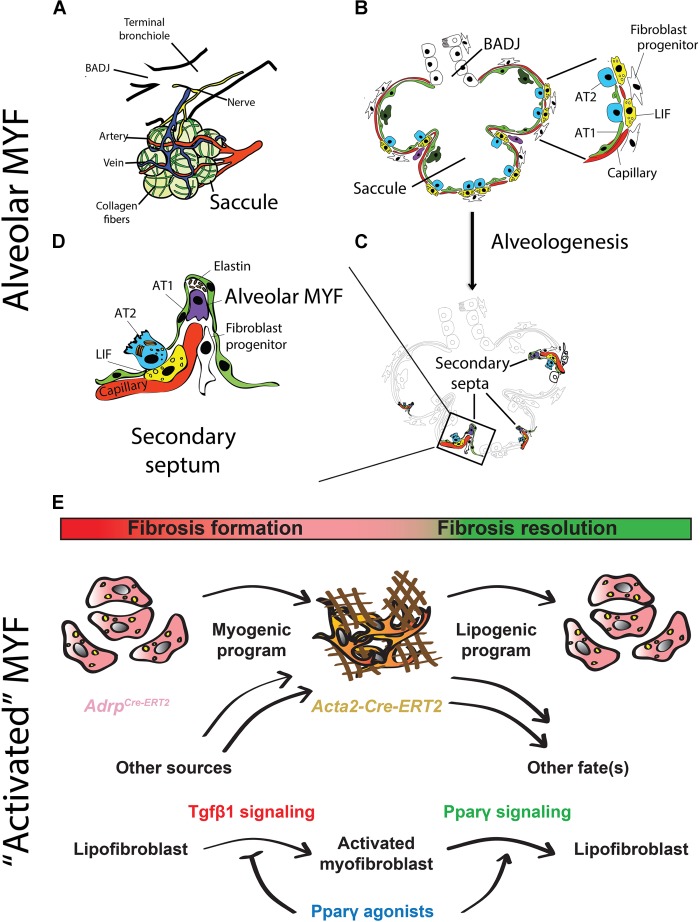
Schematic representation of alveologenesis and cell types involved. **(A)** During the saccular stage (stage preceding the beginning of alveologenesis), the lung exhibits primitive alveoli (called saccules), which are surrounded by blood vessels, collagen fibers and nerves. **(B)** The alveolar saccule in the saccular stage is characterized by the presence of AT1/2, coating the walls of the saccule, surfactant production, production of collagen and elastin by fibroblasts as well as expansion of the capillary tree. **(C)** During the alveolar stage, the lung undergoes a subdivision of the saccules by a process called “secondary septation” that will give rise to mature alveoli. **(D)** Secondary septa start to appear at the place of elastin deposition, which is produced by alveolar MYF. The septa elongate toward the alveolar sac airspace. A double layer of capillaries become thinner giving rise to a one-layer network for more efficient gas exchange. **(E)** The origin and fate of “activated” MYFs was investigated using lineage-tracing approaches. LIFs are progenitors for “activated” MYFs in lung fibrosis. Some of the labeled “activated” MYFs dedifferentiate to LIF during fibrosis resolution. Pparγ activation blocks LIF-to-MYF transdifferentiation induced by Tgfβ1 and enhances “activated” MYF-to-LIF transdifferentiation. (adapted from Chao et al., 2016; El Agha et al., 2017).

Alveolar MYFs are Acta2-positive fibroblasts present in the lung during alveologenesis, that starts postnatally in mice. These cells secrete extracellular matrix (ECM) fibers such as elastin and collagen that are required for secondary-crest formation (Vaccaro and Brody, 1978; Noguchi et al., 1989; Yamada et al., 2005; Figures 2A–D). Alveolar MYFs have been suggested to originate from Pdgfrα-positive cells. Pdgfa signaling via Pdgfrα plays a critical role in the formation of the alveolar MYFs as demonstrated by the lungs in *Pdgfa*-null newborns that suffer from the absence of alveolar MYFs and consequently display arrested alveologenesis (Bostrom et al., 1996; Lindahl et al., 1997). However, lineage tracing of Pdgfrα-positive cells during lung development is lacking in the studies mentioned above. It needs to be demonstrated that Pdgfrα-positive cells labeled early during the pseudoglandular stage are progenitors for alveolar MYFs. Postnatally, Pdgfrα is expressed by multiple mesenchymal cell types including ASMCs, alveolar MYFs, and LIFs (Ntokou et al., 2015; Li et al., 2018).

## Ectopic Mesenchymal Fgf10-Fgfr2B Autocrine Loop During the Early Pseudoglandular Stage Leads to the Absence of Alveolar MYF Formation at Birth

So far, the impact of fibroblast growth factor signaling on the differentiation of the lung mesenchyme has been poorly investigated. *Fgfr2c^+/Δ^* mice were used as an *in vivo* experimental model to investigate the function of mesenchymal Fgf signaling. These mice ectopically express Fgfr2b in mesenchymal tissues from early developmental stages and therefore display an Apert syndrome-like phenotype, which is characterized by malformations of the face, skull and feet and the respiratory system (Hajihosseini et al., 2001). Former studies have proved that early establishment of autocrine Fgf10–Fgfr2b signaling in the lung mesenchyme inhibits the formation of the SMCs as well as the alveolar MYFs and results in reduced fibronectin and elastin deposition. In addition, the branching process is impaired and the level of Fgf and canonical β-catenin signaling in the epithelium is reduced. These mutant lungs display arrested development of the terminal airways and an “emphysema like” phenotype postnatally (De Langhe et al., 2006). These results indicate that Fgf signaling represses the differentiation of the early alveolar MYF progenitors. It is not clear whether this is due to a direct effect of Fgf on the mesenchyme or to an indirect effect via the epithelium. However, Fgf9, which is the natural and major Fgf ligand acting on the mesenchyme during early lung development, is capable of repressing *in vitro* the expression of Acta2 in primary cultures of lung mesenchymal cells. This suggests that *in vivo* Fgf signaling maintains the mesenchymal progenitors undifferentiated and proliferative (del Moral et al., 2006a). A counter-intuitive result was obtained by Perl and Gale. The authors took advantage of the *Tg(Sftpc-rtTA)/+; Tg(tet(O)solFgfr2b)/+* double-transgenic mice to induce in the lung, via the administration of docycyclin, the expression of soluble Fgfr2b acting as a receptor to sequester all Fgfr2b ligands. Expression of this decoy receptor in the lung from E14.5 to E18.5 disrupts alveologenesis postnatally. Secondary-septa formation with the presence of alveolar MYFs, can be partially enhanced in this model by treating the animals with RA between P35 and P48. The effect of RA can be blocked by the concomitant re-expression of the soluble form of Fgfr2b using doxycycline. This leads to an increase in Pdgfrα-positive cells and an associated decrease in Acta2-positive cells (Perl and Gale, 2009). One possible interpretation for these results is that alveolar MYFs require Fgfr2b ligands, likely Fgf10, for their differentiation. It was also proposed that Pdgfrα-positive LIFs can transdifferentiate into alveolar MYFs but, so far, there is no lineage tracing-based evidence to support this conclusion. It was also shown that Fgfr2b ligands are required for alveolar MYFs formation during alveolar regeneration after pneumonectomy (Chen et al., 2012). However, the attenuation of all Fgfr2b ligands postnatally does not result in any lung development defect. Alveologenesis, which is characterized by the formation of secondary septa containing alveolar MYFs, occurs normally (Hokuto et al., 2003). It can therefore be concluded that normal alveologenesis does not require secreted Fgfr2b ligands.

## Characterization of the Alveolar MYFs in the Developing Lung

The analysis of the differentiation of labeled progenitor cells (using the *Fgf10^CreERT2^, Wt1^CreERT2^, Gli1^CreERT2^*, and *Axin2^CreERT2^* driver lines crossed with a fluorescent reporter line) toward the MYF and SMC lineages showed that Fgf10-positive and Wt1-positive cells displayed a minor contribution to the SMC lineages, while Gli1-positive and Axin2-positive cells significantly contributed to both the SMC and alveolar MYF lineages, but also to other lineages (Moiseenko et al., 2017). Labeling at E11.5 of Acta2-positive cells using the *Tg (Acta2-CreERT2)* transgenic line showed that these cells did not proliferate to produce new SMCs at later stages. However, if the labeling of Acta2-positive cells occured at E15.5, the labeled cells constituted most of the SMCs (85–97%) as well as the majority of the alveolar MYF progenitors in the E18.5 lungs. Gene arrays of Acta2-positive cells isolated by fluorescence-activated cell sorting allowed establishing transcriptomic signatures for airway and VSMCs vs alveolar MYF progenitors at E18.5. Interestingly, alveolar MYF progenitors expressed very high levels of *Fgf10* compared to SMCs (Fold Change = 29.04, *p* < 0.0001). It is still unclear if Fgf10 acts in an autocrine or paracrine way on alveolar MYF progenitors to sustain their differentiation (Moiseenko et al., 2017). These results will allow defining pathways potentially important for the formation of alveolar MYF.

## Evidence for LIF to “Activated” MYF Reversible Differentiation During Fibrosis Formation and Resolution

Idiopathic pulmonary fibrosis (IPF) is a form of progressive interstitial lung disease of unknown origin. As no efficient treatment is available, IPF patients exhibit a high lethality rate. In this disease, a prominent number of “activated” MYFs are present in the lung parenchyma. These cells excessively deposit extracellular matrix proteins, literally turning the lung into “a block of cement”. These changes compromise the lung architecture and function, thereby leading to impaired gas exchange. The origin of “activated” MYFs as well as the molecular mechanisms governing the formation and the fate of these cells in fibrosis formation and resolution have been investigated using specific driver lines to target different mesenchymal populations (El Agha et al., 2017; Figure 2E). Interestingly, a reversible lipogenic-to-myogenic transdifferentiation during fibrosis formation in mice has been demonstrated. In addition, in IPF lungs, compared to the donor controls, loss of LIFs and accumulation of “activated” MYFs were observed (El Agha et al., 2017). Finally, this fate switching has been validated in primary human lung fibroblasts from IPF patients, suggesting that drugs that are capable of enhancing myogenic-to-lipogenic transdifferentiation could be instrumental in treating IPF patients (El Agha et al., 2017). Interestingly, the concept of LIF to “activated” MYF transition was already proposed but not proven using modern lineage tracing approaches by several teams including Rehan and colleagues. They showed that LIFs derived from neonatal rat lungs transdifferentiated into MYF in response to hyperoxia (Rehan and Torday, 2003) as well as to nicotine exposure (Rehan et al., 2005).

## Fgf10 Expression Inversely Correlates With Disease Progression in IPF Patients

The expression levels of *FGF10* in IPF vs non-IPF donor lung tissue samples were monitored by qPCR. A significant increase in *FGF10* expression in IPF lungs compared to donor’s lungs was reported (El Agha et al., 2017). By immunohistochemisty, FGF10 expression in IPF lungs appears to be higher in highly remodeled parenchyma compared to the fibrotic foci (which are sites of early fibrotic response) (El Agha et al., 2018). It has been therefore proposed that FGF10 could be associated with either the fibrotic process or with the associated repair process. Suggesting that FGF10 is rather involved in the repair process, it was shown that FGF10 expression levels are inversely correlated with disease progression (El Agha et al., 2018). Supporting this result, mesenchymal stromal cells isolated from the bronchoalveolar lavage of patients with progressive IPF displayed less *FGF10* expression compared to corresponding cells isolated from patients with stable IPF suggesting that FGF10 deficiency could be indeed causative for disease progression (Chanda et al., 2016).

## Activation of Pparγ Signaling Antagonizes Tgfβ1-Mediated Fibrogenic Response

Peroxisome proliferator-activated receptor gamma signaling activation by rosiglitazone has been identified to antagonize Tgfβ1 activity in IPF patients and as a result decreased fibrosis formation in bleomycin-treated mice was reported (Genovese et al., 2005; El Agha et al., 2017). One potential mechanism of action of rosiglitazone is reinforcing the lipogenic phenotype at the expense of the MYF phenotype. This hypothesis is consistent with increased LIF formation in the developing lung *in vivo* following deletion of the Tgfβ1 receptor *Alk5* in the mesenchyme (Li et al., 2016). To test this possibility, human lung fibroblasts were cultured in the presence of rosiglitazone (20 mM) and/or recombinant Tgfβ1 (1 ng/mL). When the cells were treated with TGFβ1, the expression of lipogenic markers *PPAR*γ and *ADRP* were significantly inhibited while the expression of myogenic markers *ACTA2* and *COL1A1* were promoted compared to the normal cells. Rosiglitazone treatment strongly up-regulated *ADRP* expression and reduced the effect of TGFβ1. Consistent with this finding, rosiglitazone treatment significantly attenuated TGFβ1-mediated up-regulation of *ACTA2* and *COL1A1*. These results demonstrate that TGFβ1 in IPF functions by reinforcing the myogenic phenotype. Recently, it was reported that metformin, an adenosine monophosphate-activated protein kinase (AMPK) agonist used to treat diabetes was also efficient in accelerating fibrosis resolution after bleomycin-injury in mice (Rangarajan et al., 2018). It remains to be determined if metformin is also capable of accelerating the “activated” MYFs to LIFs transdifferentiation. An important question to ask is what is the impact of metformin on the LIF-AT2 interaction and on *FGF10* expression in particular: does it re-enforce this interaction? This would suggest that metformin could be used as a powerful drug to enhance lung regeneration after injury.

## Fgf10 and Other Human Lung Diseases

Fibroblast growth factor 10 haploinsufficiency in human is associated with chronic obstructive pulmonary disease (COPD). COPD is a disease characterized by major remodeling of the conducting airway epithelium as well as alterations of the respiratory epithelium. Patients with *FGF10* haploinsufficiency display a nonreversible airway obstruction ultimately resulting in the development of COPD (Klar et al., 2011). Reduction in FGF10 levels in the lungs of prematurely born infants is also associated with a disease called bronchopulmonary dysplasia, where the lungs are arrested at the saccular stage, prior to the formation of the alveoli, the mature respiratory units critical for normal lung function (Figure 1E). For more information on this topic, we refer the readers to two excellent reviews, which are part of this special issue on FGF10 in development, homeostasis, disease and repair after injury (Prince, 2018; Yuan et al., 2018).

## What Is Next for Fgf10?

Even though a plethora of knowledge has been gained on Fgf10’s mechanisms of action, interaction with different signaling pathways, and its cellular targets, progress in the field has been delayed by the fact that early *Fgf10* deletion *in vivo* leads to organ agenesis. The rapid and complete disappearance of the organ or cells of interest following *Fgf10* deletion makes it difficult, for example, to identify its primary transcriptional targets and its biological activity. Better tools, such as inducible and reversible specific decoy Fgfr2b receptors will be very useful in this context. Despite its powerful effect in promoting lung regeneration following different types of injury, the use of FGF10 in clinical trials is lagging behind. One major obstacle for its clinical use is the relatively low level of biological activity of the corresponding recombinant protein, which associates with a high affinity with heparan sulfate proteoglycans, resulting in the trapping of the protein in the extracellular matrix. Progressive loss of activity and lack of diffusion of the protein, combined with undesired side effects (such as swelling of the tongue or the eyelid and high mucus production in the gut due to goblet cell metaplasia) following systemic FGF10 intravenous delivery, have obscured potential beneficial effects. In the future, localized administration of a stable form of FGF10 should be the new *modus operandi* for successful clinical trials.

## Author Contributions

JW and XC wrote the review. CC and SB edited the review.

## Conflict of Interest Statement

The authors declare that the research was conducted in the absence of any commercial or financial relationships that could be construed as a potential conflict of interest.

## Abbreviations

Adrp: Adipose differentiation-related protein
ASMC: Airway smooth muscle cell
AT1: Alveolar type 1
AT2: Alveolar type 2
Fgf10: Fibroblast growth factor 10
Fgfr2b: Fibroblast growth factor receptor 2 b
LIF: Lipofibroblast
miR: microRNA
MYF: Myofibroblast
Pdgfrα: Platelet derived growth factor receptor alpha
Pparγ: Peroxisome proliferator-activated receptor gamma
PthrP: Parathyroid hormone related protein
RA: Retinoic acid
SHF: Second heart field
Tgfβ1: Transforming growth factor beta 1
VSMC: Vascular smooth muscle cell
